# Therapeutic efficacy of Artemether/Lumefantrine (Coartem^®^) against *Plasmodium falciparum *in Kersa, South West Ethiopia

**DOI:** 10.1186/1756-3305-3-1

**Published:** 2010-01-05

**Authors:** Ashenafi Assefa, Moges Kassa, Gemechu Tadese, Hussen Mohamed, Abebe Animut, Tesfayae Mengesha

**Affiliations:** 1 Ethiopian Health and Nutrition Research Institute, PO Box: 1242, Addis Ababa, Ethiopia; 2 Aklilu Lema institute of Patho-biology, Addis Ababa University, PO Box: 1176, Addis Ababa, Ethiopia

## Abstract

**Background:**

Artemether/Lumefantrine (Coartem^®^) has been used as a first-line treatment for uncomplicated *Plasmodium falciparum *infection since 2004 in Ethiopia. In the present study the therapeutic efficacy of artemether/lumefantrine for the treatment of uncomplicated *P. falciparum *infection at Kersa, Jima zone, South-west Ethiopia, has been assessed.

**Methods:**

A 28 day therapeutic efficacy study was conducted between November 2007 and January 2008, in accordance with the 2003 WHO guidelines. Outcomes were classified as early treatment failure (ETF), late clinical failure (LCF), late parasitological failure (LPF) and adequate clinical and parasitological response (ACPR).

**Results:**

90 patients were enrolled and completed the 28 day follow-up period after treatment with artemether/lumefantrine. Cure rate was very high, 96.3%, with 95% CI of 0.897-0.992 (PCR uncorrected). Age-stratified data showed adequate clinical and parasitological response (ACPR) to be 100% for children under 5 and 97.4% and 87.3% for children aged 5-14, and adults, respectively. There was no early treatment failure (ETF) in all age groups. Fever was significantly cleared on day 3 (P < 0.05) and 98% of parasites where cleared on day 1 and almost all parasites were cleared on day 3. 72.5% of gametocytes were cleared on day 1, the remaining 27.5% of gametocytes were maintained up to day 3 and total clearance was observed on day 7. Hemoglobin concentration showed a slight increase with parasitic clearance (P > 0.05). No major side effect was observed in the study except the occurrence of mouth ulcers in 7% of the patients.

**Conclusions:**

The current study proved the excellent therapeutic efficacy of artemether/lumefantrine in the study area and the value of using it. However, the proper dispensing and absorption of the drug need to be emphasized in order to utilize the drug for a longer period of time. This study recommends further study on the toxicity of the drug with particular emphasis on the development of oral ulcers in children.

## Background

Early diagnosis and treatment of cases are the most important strategies for the control and prevention of malaria. It is crucial for proper management of the disease and to prevent further complications. *P. falciparum *has developed resistance to nearly all antimalarials in current use, although the geographical distribution of resistance to any single antimalarial drug varies greatly [1]. World maps that depict countries as having or not having drug-resistant malaria are potentially misleading, as there is great heterogeneity within countries and across political boundaries [1].

In Ethiopia, the increased resistance of *P. falciparum *to chloroquine (CQ) and sulfadoxine-pyrimethamine (SP) necessitated a change as first-line antimalarial drug for the treatment of *P. falciparum*. Consequently, Artemether/Lumefantrine (Coartem^®^) (AL) was adopted in 2004 [2,3]. Currently Coartem^® ^is being used as the first-line drug for the treatment of uncomplicated malaria [3]. A base-line study in 2004 [4] showed that AL was a highly efficacious drug with a treatment success of 99.1% and with no report of adverse effects [4].

However, the trend of malaria changes over time, and thus the cure rate, tolerance, compliance and safety of AL (Coartem^®^) need be monitored with particular emphasis to monitoring its therapeutic efficacy [2-5]. Therefore, close monitoring of resistance patterns would be required to maximize the therapeutic benefits of this drug as well as policy development and mapping of resistance areas. Based on this notion, this study set the objective to determine the efficacy of AL (Coartem^®^) in the routine treatment of uncomplicated *P. falciparum *malaria at Serbo health center, Kersa district, south west Ethiopia.

## Materials and methods

### Study Area and Population

**Figure 1:**
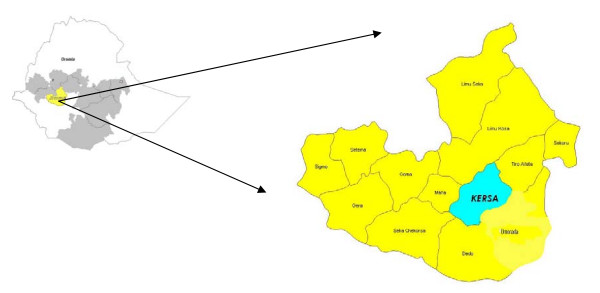
**Map of Kersa district (Wereda), Oromia, Jima zone**.

The study was conducted at Serbo Health Center, Kersa district which is located in Jima zone, Oromia region, 323 km south west of Addis Ababa (Fig. 1) between November 2007 and January 2008. The district is malarious and covers 678.6 km square, with an altitude ranging between 1600-2400 above sea level. Malaria is the most prevalent seasonal disease in the area, accounting for 77.1% of all the reported diseases in the health center in 2006 and 2007. October to December is the peak transmission season. Both *P. vivax *and *P. falciparum *exist in the area with *P. vivax *prevailing all year, even in non-transmission (relapse) seasons.

### Study subjects

The study subjects were recruited from febrile patients visiting Serbo Health Center based on WHO inclusion guideline for the assessment and monitoring of antimalarial drug efficacy for the treatment of uncomplicated falciparum malaria [2].

### Subject inclusion criteria

The following inclusion criteria were used for the study: Mono-infection with *P. falciparum*, a parasitemia level of 1000 - 100,000/μl, absence of danger signs or signs of severe and complicated malaria according to the definition given by [2] presence of axillary temperature (> 37.5°C), absence of other concomitant infections like pneumonia which can cause fever, no use of antimalarial drug two weeks prior to the study and compliance for successive visits as per the protocol for uncomplicated malaria recommendations.

### Exclusion criteria

Patients with: (i) severe signs of malaria according to WHO criteria, which included severe anaemia defined by haemoglobin < 5 g/dl, (ii) history of allergic reactions to the study drug Coartem^® ^(iii) concomitant presence of febrile condition with the potential to confound study outcome (Example: acute respiratory infection (ARI), measles, severe diarrhea, etc.) (iv) severe malnutrition and (v) pregnant and lactating women were not included in the study [2].

### Sample collection

Finger-prick blood samples were collected from consenting patients for malaria parasite identification and hemoglobin level measurement. Patients that satisfied the criteria, were enrolled to the study and followed up on days 1, 2, 3, 7, 14, 21, and 28 where finger-prick samples were taken for microscopic glass slides. A parallel drop of blood was collected on filter paper on day 0 during enrollment and on any unexpected visit. The filter paper was air dried and stored in a self sealing plastic bag with desiccators for further molecular analysis.

### Sample size

Considering the very low or unknown proportion of clinical failure of artemether/lumefantrine resistance in Ethiopia, sample size was calculated with 10% precision, 95% confidence interval, 15% withdrawal and 28 day study period, 90 study participants were included in the study [2].

### Microscopic diagnosis

Thick and fixed thin blood smears were stained with 10% Giemsa (pH 7.4) for ten min. Blood films were taken at least eight times for each patient during the study period (day 0, 2, 3, 7, 14, 21 and 28) and on any unexpected visit by the patient. These smears were used in species identification and thick blood films were used to determine parasite density (100 high power field (HPF) were the number of asexual parasites per HPF were recorded, according to the method described in the WHO Protocols [2-6]. A blood slide was considered negative when no parasites were seen after examining 100 fields. Parasite count was based on the number of asexual parasites observed against 200 leukocytes. This number was then multiplied by 40 to gain an approximate count per microlitre.

Slides were read by two senior microscopists from the Ethiopian Health and Nutrition Research Institute (EHNRI) and one from Serbo health center. In case of disagreement the majority result was taken. All slides were properly documented for quality control.

### Hemoglobin measurement

Finger-pick blood sample was used to measure hemoglobin using a portable spectrophotometer (Haemocue).

### Treatment and follow up schedule

Artemether-lumefantrine (Coartem^®^) was obtained from Novartis pharma AG, Basel, Switzerland, Bach No. T2005-59, through the WHO office, Addis Ababa. All eligible patients were treated with Coartem^® ^at day 0. Dosing was a six dose regimen given twice daily for three days. Study medication was administered based on weight; the first dose was given under direct observation. The successive day's doses were given to the patient/guardian for self administration in front of health professionals in the area (Health extension workers). Patients were followed for 30 min post treatment and if vomiting occurred, a second full dose was administered. If repeated vomiting occurred, patients were withdrawn from the study. Patients were asked to return to the clinic on days 1, 2, 3, 7, 14, 21, and 28 or were highly encouraged to return whenever they did not feel well. Patients, who failed to come to the clinic at the scheduled time, were visited in their home on the same day and the necessary blood sample, reports of adverse effects and temperature data were collected.

### Patient withdrawal

Patients were withdrawn from the study in case of (i) vomiting the drug twice, (ii) withdrawal of consent, (iii) onset of a serious febrile illness, (iv) intake of any drug with antimalarial properties, (v) missing repeated treatment doses, (vi) mixed species parasitemia or (vii) any protocol violation. Patients who missed follow-up visits and did not come on the successive day despite tracing were considered as lost to follow-up. Patients withdrawn and with complications were referred to the health centers for proper treatment. Patients withdrawn for the re-appearance of *P. falciparum *were treated with quinine and those infected with *P. vivax *were treated with chloroquine.

### *In vivo *analysis and classification response

Patients were classified as early treatment failure (ETF), late clinical failure (LCF), late parasitological failure (LPF) or adequate clinical and parasitological response (ACPR) as per WHO definitions [2].

#### ETF

Development of danger signs for severe malaria on Day 1, Day 2 or Day 3, in the presence of parasitemia; parasitemia on Day 2 higher than Day 0 count irrespective of axillary temperature; parasitemia on Day 3 with axillary temperature ≥ 37.5°C; parasitemia on Day 3 ≥ 25% of count on Day 0.

#### LCF

Development of danger signs for severe malaria after Day 3 in the presence of parasitemia, without previously meeting any of the criteria of ETF. Presence of parasitemia and axillary temperature >37.5°C (or history of fever) on any day from Day 4 to Day 28, without previously meeting any of the criteria of ETF.

#### LPF

Presence of parasitemia on any day from Day 7 to Day 28 and axillary temperature > 37.5°C, without previously meeting any of the criteria of early treatment failure or late clinical failure.

#### ACPR

Absence of parasitemia on Day 28 irrespective of axillary temperature without previously meeting any of the criteria of ETF, LTF or LPF.

### Molecular analysis

Samples that were classified as ETF, LCF or LPF were genotyped to differentiate recrudescence from re-infection. Blood samples for PCR were collected on standard filter paper, air dried and stored in cool and dark boxes with desiccants. Genotypic analysis was performed at EHNRI as previously described for msp-1 [7] and msp-2 [8,9]. Cases in which pre- and post-treatment genotypes were identical were considered as recrudescence; cases in which pre- and post-genotypes were different were considered as re-infection, mixed genotypes were classified as failures.

### Ethical clearance

The study protocol was reviewed and approved by Ethical Review Committee of Ethiopian Health and Nutrition Research Institute and Nation Ethical Review Committee of Ethiopia.

### Consent form

Prior to the trial, a consent form was signed by the patient or by the parent/guardian after being translated and read in the vernacular language that the patients or the carers understood.

### Statistical Analysis

All the data from recruited patients were imported into an Excel spreadsheet and the WHO designed Excel data analysis program was used for analysis and SPSS 11 were used for descriptive statistics and comparing data [2].

## Results

During the study, 1628 febrile cases, clinically suspected to be infected with malaria, were screened for eligibility. Of these 428 (26.3%) had malaria-positive slides and 361 (22.2%) of them had *P. falciparum *mono-infections. Of the *P. falciparum *mono-infected patients, 236 could not be enrolled in the study for having a parasite load below 1000 parasites per microliter of blood. Of the remaining 125 patients who were eligible for the study, 35 were excluded for the following reasons: 27 were out of the reachable area, 2 were pregnant, another 4 took anti-malaria drugs before commencement of the study and the remaining 2 refused consent.

**Figure 2:**
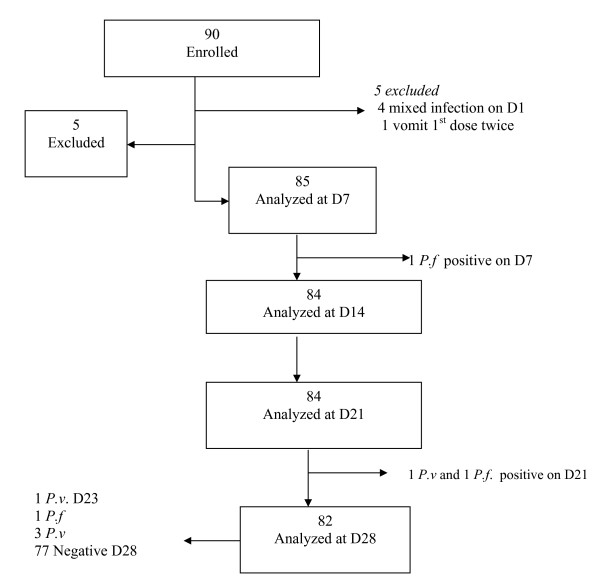
**Details on study inclusion and follow up progress**. *P.f *= *P. falciparum*; *P.v *= *P. vivax*.

Finally 90 patients fulfilled all the criteria and enrolled in the study (Figure 2). Five were excluded for vomiting the first dose twice and having mixed infection. On day 7, 85 slides from patients were examined and one person was found to be *P. falciparum *positive and excluded as treatment failure and treated with quinine. On day 21, 84 slides were examined and two positive slides were found, one positive for *P. falciparum *and the other for *P. vivax*. The first was removed as treatment failure and the second withdrawn from the study and treated with chloroquine. On the final day (Day 28), slides from the remaining 82 patients were examined and one *P. falciparum *and three *P. vivax *cases were recorded. The first was considered a treatment failure and treated with quinine, and the later three were taken as negative slides for data analysis and were treated with chloroquine (Figure 2.)

The base-line characteristics of the 90 eligible patients are given in Table 1. Forty eight (53%) of the patients were females with 1.14 female to male ratio. The age stratification showed 33 (37%) were under 5, 41 (46%) between 5 and 14 and the remaining 16 (17.8%) were adults (above 14). Auxiliary temperature was comparable in the three age groups with mean of 38 ± 0.3°C at enrollment. Paracetamol was given for all patients with auxiliary temperature greater than or equal to 38°C. Hemoglobin concentration increased with age, ranging from 5.5 to 17.1 regardless of parasite load. Parasitic load ranged from 1000-100,000 in all age groups and the highest mean parasite density was observed in the 5-14 age group followed by under 5 and adults. Gametocytes at enrollment were observed in 6/90 (6.7%) patients and declined with an increase in age, 9% in under 5, 7.3% in 5-14 and none in adults.

**Table 1:** Base line characteristics

	< 5 years	5-14 years	≥ **15 years**	Total
				
	n = 33	n = 41	n = 16	n = 90
Mean age (range)	2.8 (1-4.5)	9.1 (15-14)	21.3 (15-30)	9 (1-30)
Male	15 (45.5%)	20 (48.8%)	7 (43.8%)	42 (46.7%)
Weight(kg)	11.7	23.2	48.4	23.5
Temp (°C)	38.3(36.9-40.6)	38 (35.9-40.9)	38 (36.9-39)	38.1 (35.9-40.9)
Hemoglobin (g/dl)	11.13 (5.5-15.5)	12.5 (7.7-15.5)	13.6 (9.4-17.1)	12.2 (5.5-17.1)
Mean Parasite density per μl	18,983	32,607	5,541	22,660
Gametocyte carriage (n)	9% (3)	7.3%(3)	0%	6.7% (6)

In the present study 7.7%(7/90) patients (PCR uncorrected) were excluded during follow-up. And thus a total 82 patients were analyzed at the end-point (day 28) and the result was satisfactory compared with other studies. All patients completed the study properly and none of them missed the follow-up.

Cure rate was very high in all age groups (Table 2). The overall cure rate was 96.3% with 95% CI of 0.897-0.992 (97.5% PCR corrected). ACPR was 100% in the children under 5 and 97.4% (100% PCR corrected) and 87.3% in the 5-14 and adult groups, respectively. There was no ETF in all age groups. There was one LCF (1.2%), in the adult group and one LPF (PCR uncorrected) for each of the 5-14 and adult groups with the overall rate of 2.4%. However, the overall rate reduced to 1.2% as PCR adjustment ruled out one of the parasite re-appearance in the 5-14 age group as re-infection.

**Table 2:** PCR corrected and uncorrected cure rate of day 28 analysis stratified by age

	*Under 5 (n = 33)*			*5-14(n = 41)*				*Adult (>14)(n = 16)*			*Total (n = 90)*			
				
	*No.*	*% Prev. PCR uncorr.*	*% Prev. PCR corre.*	*No.*	*% Prev. PCR uncorr.*	*No.*	*% Prev. PCR corre.*	*No*	*% Prev. PCR uncorr.*	*%Prev. PCR corre.*	*No.*	*%Prev. PCR uncorr.*	*No.*	*% Prev. PCR corre.*
*ETF*	0	0.0	0.0	0	0.0	0	0.0	0	0.0	0.0	0	0.0	0	0.0
*LCF*	0	0.0	0.0	0	0.0	0	0.0	1	6.3	6.3	1	1.2	1	1.2
*LPF*	0	0.0	0.0	1	2.6	0	0.0	1	6.3	6.3	2	2.4	1	1.2
*ACPR*	28	100.0	100.0	37	97.4	37	100.0	14	87.3	87.3	79	96.3	79	97.5
*Total Analysis*	28			38		37		16			82		81	
*With.*	5			3		4		0			8		9	
*Loss*	0	15.2	15.2	0	7.3	0	9.8	0	0.0	0.0		8.9	0	100.0

*Total*	33			41		41		16			90		90	

ETF: Early treatment failure

LCF: Late clinical failure

LPF: Late parasitological failure

ACPR: Adequate clinical and parasitological response

With: withdrew

Prev: Prevalence

Corre: corrected

Uncorr: Uncorrected

No: Number

%: percent

**Figure 3:**
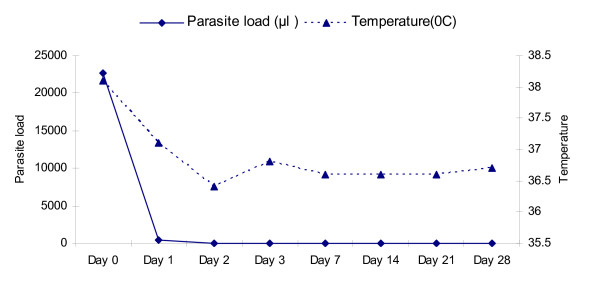
**Summary of the average body temperature (°C) and parasite density (μl) over the study period**.

As shown in Figure 3, fever clearance was similar in all age groups, fever was significantly cleared on day 3 in all patients (P < 0.05). 98% of parasites were cleared on day 1 and almost all parasites where cleared on day 3. In adults and 5-14 age groups, parasites were totally cleared from blood on day 2 and 3, respectively. In under 5 patients, a small fraction (1.1%) of patients showed parasite occurrence on day 3 and total clearance occurred on Day 7 (Table 3).

**Table 3:** Summary of the average body temperature (°C), and parasite load (μl) stratified by age.

	*Under 5 (n = 33)*		*5-14 (n = 41)*		*Adults (n = 16)*	
			
Day	Temp	Para	Temp	Para	Temp	Para
D0	38.3	18983	38	32607	37.9	5541
D1	37.4	350.2	37	676	37	102
D2	37.3	13.3	36.6	59.8	36.5	0
D3	37	1.1	36.6	0	36.7	0
D7	36.9	0	36.5	0	36.4	0
D14	37	0	36.4	0	36.6	0
D21	36.9	0	36.5	0	36.3	0
D28	37	0	36.5	0	36.4	0

Temp: Temperature

Para: parasite load (μl)

**Figure 4:**
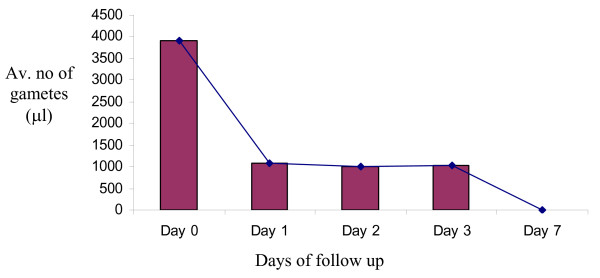
**Gametocytemia after treatment**.

Gametocyte clearance declined with age in the early treatment days, 72.5% were cleared on day 1. However, the remaining, about 28% of gametocyte load was maintained up to day 3 irrespective of treatment. However, total clearance was observed on day 7 (Figure 4).

Hemoglobin concentration showed a slight increase with parasitic clearance from the blood, however, the mean change in hemoglobin concentration was not statistically significant between day 0, day 14 and day 28 (P > 0.05) (Table 4). Although improvement was observed in hemoglobin concentration with decline in parasite density, the correlation between the two was statistically insignificant (p = 0.132).

**Table 4:** Summary of the average hemoglobin concentration (mg/dl) stratified by age.

Days/Age	Under 5 (n = 33)	5-14 (n = 41)	Adults (n = 16)
			
	Hb	Hb	Hb
D0	11.13	12.5	13.6
D14	11.6	12.2	13.3
D28	11.7	12.8	13.9

P Value	> 0.05	> 0.05	> 0.05

Overall, there were 255 adverse events reported upon taking the drug (data not shown) of which the most common ones were cough in 46% of cases, headache in 17%, anorexia in 9%, weakness/fatigue in 7%, abdominal pain in 5%, diarrhea and sleep disorder in 3%, and vomiting in 2% of the cases. There was also a considerable occurrence of inflammation of the oral mucosa (mouth ulcer) in 7% of patients.

Most of adverse events disappeared with resolution of the disease, however cough prevailed after treatment. The occurrence of mouth ulcer (peri-oral wound) is not a commonly reported adverse effect of Coartem^®^, however, it was recorded in considerable number of patients (7%) in under 5 and 5-14 age groups.

## Discussion

Our findings have indicated that artemether/lumefantrine (Coartem^®^) has a high cure rate. The results showed very high efficacy in the very young children less than five years of age followed by middle age children and adults. Results from the present study were consistent with previous studies conducted in Ethiopia and elsewhere [4,10-16]. The overall cure rate was 96.3% (97.5% PCR corrected). Although the results obtained in the present study are similar to the results of a study conducted in Uganda [15] the age-stratified results from Uganda showed a reversed trend i.e., 100%, 97.7% and 96.4% cure rates were obtained for adults, 5-14 and under five, age groups, respectively. This may be due to differences in malaria exposure, where adults in more endemic areas acquire immunity to enhance the drug effect.

Inclusion of all age groups in the trial was because the area showed unstable malaria of lower (hypo) endemicity where inhabitants were considered to be non-immune to malaria. In accord with this, the child splenomegally rate of the district was found to be 7.3% (data not shown) confirming the hypo-endemicity of the study site. Hence our study area being one of an unstable seasonal malarious type, no such stratified immunity acquisition was expected. However, the high cure rate among children could be due to higher milk/fat content in their diet, which facilitates the absorption and hence efficacy of Coartem^®^. This is actually a known phenomenon with lipophilic antimalaria drugs given in the oral route [17]). Nevertheless, the age differences in cure rate were low.

Of the total of the three treatment failures (one LPF and two LCFs), PCR genotyping showed two of the three recurrent infections (one LPF and one LCF) were recrudescence and one of the LCF was PCR unresolved and excluded. The PCR analysis result increased the overall efficacy in to 97.5%, and the recrudescence rate with Coartem^® ^was found to be very low at about 2.5%.

In this study, although Coartem^® ^clears fever and parasitemia in a very short period of time (less than three days), the concentration of hemoglobin fails to increase within the same period of time. Increments in hemoglobin level have been noted with clearance of fever and parasitemia, especially in those patients who had a lower level of hemoglobin concentration in the baseline of treatment than others. However, the difference was statistically insignificant (p < 0.05). Similar findings were also observed in other studies [10,14]. The high prevalence of geohelminths (especially hookworm) in the area can also be a contributing factor to the chronicity of anemia. A recent study in Asendabo, one of the nearby districts of Jima zone, showed 27.6% (102/370) of the sample population to be anemic, of which, 26.5% (27/102) had hookworm infection, 19.6% (20/102) were malaria positive and 15.8% (19/102) were physiologically anemic with no infection and 22.5% with co-infection. Anemia was significantly higher in hookworm positive individuals [18].

Fever is a manifestation of malaria that most frequently causes discomfort. Thus, it was encouraging that patients treated with Coartem^® ^become afebrile within the first two or three days. An antipyretic (paracetamol) was given for febrile patients (body temperature above 38°C), however the number of patients who required paracetamol during the follow up period was significantly low compared to the baseline. A similar finding was noted in other studies [4,10-15,17,19]. This is due to the inherent feature of Coartem^®^, as a drug with fast resolution capacity of clinical symptoms.

The study showed the decline of gametocytes with treatment. They were almost cleared from the patients after seven days. This rate of clearance was higher when compared with other studies, which reported the presence of gametocytes up to the 14^th ^day and beyond [19,14]. It has been reported that artemesinin derivatives are gametocidal [10,20,21] and the absence of gametocytes, seven days post treatment with Coartem^® ^supports the report that there was some effect of the artemisinin derivative on the sexual stage of the parasite [21]. Other aretemesinin combinations (ACTs) were also reported to have the same effect [18,14]. Coartem^® ^decreases gametocytes by 6-8 fold compared with SP and CQ [21]. Thus, the present finding is in line with other studies. In other studies, Coartem^® ^was reported to reduce gametocyte infectivity down to 40% [21] of paramount importance in the control of transmission compared with other drugs.

Studies showed that the overall toxicity of artemether combinations is very low, the drug is well-tolerated and non-compliance is very low [11,17,21]. A number of side effects were seen for Coartem^® ^most of which are similar to the symptoms of malaria itself. An adverse effect noted in 16.5% of all patients was dry, unproductive cough at baseline. This appears to be very likely a symptom associated with the disease. Interestingly, a further 45% of all patients developed this symptom after commencement of treatment. Similar results were reported in a study conducted in Uganda where, 37% and 40% of cough was observed before and after treatment, respectively [13]. However cough, headache, abdominal pain and anorexia were the most common pro-dromal complaints reported. In addition, the present study revealed the development of oral inflamation (oral ulcer) with improved clinical and parasitological signs, indicating that it is not hyper-pyrexia induced phenomena. To the best of our knowledge these side effect has not been reported for Coartem^® ^and its etiology is not clear. Low levels of immunity due to concurrent diseases such as HIV could be one reason. But, the problem has been noted in 7.1% (6/85) of the patients. The clinicians in the area have also reported the same side effect of the drug in the past. It is therefore, very unlikely to consider the occurrence to be due to chance.

## Conclusion and Recommendation

This study has proved the excellent therapeutic efficacy profile of the combination drug, given as a three day multiple regimen of the optimized formulation for the treatment of *P. falciparum *in Kersa district south west, Ethiopia. Four years after its introduction as a first line drug, Coartem^® ^has been proved to be effective, in rapidly clearing fever and parasites within 72 h in patients suffering from uncomplicated malaria. However, the proper absorption of the drug must be ensured and monitored through controlled nutritional interventions so that the parasites are not exposed to mal-absorbed low, tolerable level of the drug.

This work also recommends further study on blood concentration of the drug and pharmaco-vigilance on the drug toxicity particularly the development of mouth ulcer in children, as drug adverse effects hamper compliance to the proper dosage which in turn enhances development of drug resistance.

## Competing interests

The authors declare that they have no competing interests.

## Authors' contributions

AA was fully involved in all phases of the study, including data collection and monitoring both in the field and in laboratory during data analysis, interpretation, and writing the manuscript; MK designed the study project, supervised the study and revised the manuscript. GL and HM were involved in field data collection and molecular analysis. AA^2 ^involved in preparing the manuscript. TM involved in data analysis, interpretation and critical revision of the manuscript for publication. All authors read and approved the final manuscript.
